# Meristem micropropagation of cassava (*Manihot esculenta*) evokes genome-wide changes in DNA methylation

**DOI:** 10.3389/fpls.2015.00590

**Published:** 2015-08-13

**Authors:** Shedrack R. Kitimu, Julian Taylor, Timothy J. March, Fred Tairo, Mike J. Wilkinson, Carlos M. Rodríguez López

**Affiliations:** ^1^Plant Research Centre, School of Agriculture Food and Wine, Faculty of Sciences, University of AdelaideAdelaide, SA, Australia; ^2^Biometry Hub, School of Agriculture Food and Wine, Faculty of Sciences, University of AdelaideAdelaide, SA, Australia; ^3^School of Agriculture Food and Wine, Faculty of Sciences, University of AdelaideAdelaide, SA, Australia; ^4^Mikocheni Agricultural Research InstituteDar es Salaam, Tanzania

**Keywords:** methylation sensitive GBS, genotyping by sequencing, micropropagation, cassava, somaclonal variation, DNA methylation, epigenetics, methylation-sensitive amplified polymorphisms

## Abstract

There is great interest in the phenotypic, genetic and epigenetic changes associated with plant *in vitro* culture known as somaclonal variation. *In vitro* propagation systems that are based on the use of microcuttings or meristem cultures are considered analogous to clonal cuttings and so widely viewed to be largely free from such somaclonal effects. In this study, we surveyed for epigenetic changes during propagation by meristem culture and by field cuttings in five cassava (*Manihot esculenta*) cultivars. Principal Co-ordinate Analysis of profiles generated by methylation-sensitive amplified polymorphism revealed clear divergence between samples taken from field-grown cuttings and those recovered from meristem culture. There was also good separation between the tissues of field samples but this effect was less distinct among the meristem culture materials. Application of methylation-sensitive Genotype by sequencing identified 105 candidate epimarks that distinguish between field cutting and meristem culture samples. Cross referencing the sequences of these epimarks to the draft cassava genome revealed 102 sites associated with genes whose homologs have been implicated in a range of fundamental biological processes including cell differentiation, development, sugar metabolism, DNA methylation, stress response, photosynthesis, and transposon activation. We explore the relevance of these findings for the selection of micropropagation systems for use on this and other crops.

## Introduction

Epigenetic control of gene expression plays an important role in development (Meyer et al., 2013). Indeed, normal development in complex higher organisms is dependent upon both spatial and temporal control of gene expression (Zhang et al., 2010), much of which is facilitated by dynamic operation of various epigenetic regulatory systems (Morgan et al., 2005). DNA methylation, and more specifically cytosine methylation (i.e., the incorporation of a methyl group to carbon 5 of the cytosine pyrimidine ring to form 5-methylcytosine) is present across many eukaryotic phyla, including plants, mammals, birds, fish, and invertebrates and provides an important source of epigenetic control for gene expression (Su et al., 2011). In plants, cytosine methylation can occur in three motif contexts (CG, CHG, or CHH, where H = a nucleotide other than G; Rodríguez López and Wilkinson, 2015). DNA methylation occurring within promoters or coding regions typically act to repress gene transcription and can be evoked by small interfering RNA-directed DNA Methylation (RdDM; see Matzke et al., 2007; Verdel et al., 2009). *De novo* DNA methylation directed by RdDM has been implicated in various types of plant stress responses (e.g., Agorio and Vera, 2007; Tricker et al., 2012, 2013) and developmental progression (e.g., Ruiz-García et al., 2005; Kinoshita et al., 2007).

The vegetative multiplication of elite genotypes to generate the large numbers of plants necessary for commercial production is an essential element for the commercial cultivation of all clonal crops and also of many perennial seed crops. The deployment of *in vitro* propagation methods rather than more traditional propagation approaches (such as field cuttings) can greatly increase the clonal multiplication throughput (Robert et al., 1992; Quiroz-Figueroa et al., 2006) and so accelerate the time to production. However, some *in vitro* multiplication techniques are associated with high levels of (usually) unwanted variability; known collectively as ‘somaclonal variation’ (e.g., Peraza-Echeverria et al., 2001). These aberrant regenerated plants can arise from both genetic and/or epigenetic-mediated alterations to gene expression and have sometimes led to significant economic losses. For example, around 5% of commercial oil palm (*Elaeis oleifera*) plants regenerated via somatic embryogenesis bore somaclonal abnormalities that included the mantled inflorescence syndrome (Jaligot et al., 2000). The appearance of these off-types was later associated with changes to their global DNA methylation status (Matthes et al., 2001) and linked to the use of specific plant hormones, growth regulators and nutrients in the culture media (Varga et al., 1988; Morcillo et al., 2006). The nature of the *in vitro* propagation system used to produce regenerated plants can have a profound effect on the likelihood of producing significant quantities of somaclonal variant plants. *In vitro* propagation systems that pass through an intermediate callus phase (such as in somatic embryogenesis) and so rely on a two stage process to generate new plants, are especially prone to evoking genetic and epigenetic change among the regenerated plants (Miguel and Marum, 2011). First, cells from the explant material must de-differentiate to form unspecialized callus cells. Second, some of these callus cells must re-differentiate in a manner that allows for the creation of the specialized cells needed to form tissues and organs. It appears that in some cases at least, one or both of these processes is incomplete. Certainly, Rodríguez López et al. (2010a) showed that the *C*-methylation profiles of leaves from plants recovered from somatic embryogenesis in cocoa (*Theobroma cacao*) retained many of the features of the explant tissue (staminoids) as well as only some of those found in the leaves of the mother plant. This finding suggests that at least the epigenetic DNA methylation landscape (and therefore the global gene regulation patterns) had not been entirely wiped (de-differentiated) in the callus cells prior to the formation of new adventitious plant tissues.

Induced changes to DNA methylation and associated perturbations to gene expression has been reported for genes associated with organogenesis (De-La-Peña et al., 2012) and other developmental processes in plants (Nic-Can et al., 2013). In comparison, *in vitro* regeneration protocols that preserve meristem anatomy and function, and which generate new plants from the activation of previously dormant meristems are known as micropropagation systems and are widely viewed as being genetically analogous to field cuttings. These systems are thought to generate daughter ramet plants that are morphologically and genetically faithful replicates of the original explant material (Kahn, 2012). To date, however, little is known about the degree to which the epigenetic profiles (and so associated cell regulatory processes) of regenerated plants from micropropagation represent faithful replicates of the original mother plant. In this study we combine methylation-sensitive amplified polymorphism (MSAP) and methylation-sensitive genotype by sequencing (msGBS; Xia et al., 2014) to assess the epigenetic fidelity of meristem micropropagation and to seek specific methylation signatures associated to *in vitro* propagation in cassava (*M. esculenta*).

## Materials and Methods

### Plant Material

Five varieties of cassava (*M. esculenta* Crantz) namely Kiroba, Kizimbani, Kibandameno, Mfaransa, and Mzungu were used in this study. *In vitro* micropropagated samples were obtained from the tissue culture facility of the Mikocheni Agriculture Research Institute (MARI) Dar es Salaam, Tanzania and were propagated as described by (Konan et al., 1997). Field samples of the same genotypes were grown at the Sugarcane Research Institute-Kibaha (SRI-KIBAHA), Tanzania. Samples were collected from young leaves (last leaf emerged from bud), newly mature leaves (first fully expanded leaf) and primary root tips from three individual plants representing each cassava variety both from field cutting and meristem culture samples. All samples were kept in dry ice in the field and stored at -80°C until required for DNA extraction.

### DNA Isolation

DNA was extracted from all samples at MARI using a DNeasy plant mini kit (Qiagen) according to the manufacturers’ instructions. DNA concentration and quality was estimated using a Nano-Drop 1000 Spectrophotometer (Thermo Scientific). DNA was lyophilised prior to transport to the Plant Research Centre in Adelaide, Australia for use in subsequent MSAP or msGBS analyses. Upon arrival, all DNA samples were re-suspended in nuclease free water (Sigma), and re-quantified using the Thermo Scientific NanoDrop^TM^ 1000 Spectrophotometer. DNA concentrations were standardized to produce working solutions of 10 or 20 ng/ul.

### Methylation-Sensitive Amplification Polymorphism Procedure

A modification of the MSAP technique (Reyna-Lopez et al., 1997) was used as described by Rodríguez López et al. (2012). In brief, genomic DNA was digested with a combination of the methylation insensitive restriction enzyme *EcoR*I and one of two isoschizomer enzymes that exhibit differential sensitivity to DNA methylation (*Hpa*II and *Msp*I; **Table 1**). Adapters were ligated to the digested gDNA and then used as template for the first of two consecutive selective PCR amplifications in which the primers were complementary to the adaptors but possessed unique 3′ overhangs (**Table 1**). *Hpa*II/*Msp*I selective primers were end labeled using a 6-FAM reporter molecule for fragment detection using capillary electrophoresis. A total of six primer combinations (**Table 1**) were tested in a pilot study using eight randomly selected DNA samples.

**Table 1:** Primer sequences used for MSAP.

Oligo name	Function	Sequence
Ad *Hpa*II/*Msp*I	Reverse Adaptor	GACGATGAGTCTAGAA
Ad. *Hpa*II/*Msp*I	Forward Adaptor	CGTTCT AGACTCATC
Ad. *EcoR*I	Reverse Adaptor	AATTGGTACGCAGTCTAC
Ad *EcoR*I	Forward Adaptor	CTCGTAGACTGCGTACC
Pre. *EcoR*I	Preselective primer	GACTGCGTACCAATTCA
Pre. *Hpa*II/*Msp*I	Preselective primer	GATGAGTCCTGAGCGGC
*EcoR*I5^∗^	Selective primer	GACTGCGTACCAATTCACA
*EcoR*I10	Selective primer	GACTGCGTACCAATTCAGC
*Hpa*II 2.2^∗^	Selective primer	GATGAGTCCTGAGCGGCC
*Hpa*II 2.3	Selective primer	GATGAGTCCTGAGCGGCG
*Hpa*II 2.4	Selective primer	GATGAGTCCTGAGCGGCT

*^∗^Indicates the selective primers used to analyze all samples*.

### Sample Fractionation by Capillary Electrophoresis

Single base resolution separation of the MSAP products was achieved by capillary electrophoresis on an ABI PRISM 3130 (Applied Biosystems, Foster City, CA, USA) housed at the Australian Genome Research Facility Ltd, Adelaide South Australia. Sample fractionation was performed as follows: 2 μl of the labeled MSAP products were combined with 15 μl of HiDi formamide (Applied Biosystems, Foster City, CA, USA) and mixed with 0.5 μl of GeneScan^TM^ 500 ROX^TM^ Size Standard (Applied Biosystems, Foster City, CA). Samples were heat-denatured at 95°C for 5 min and snap-cooled on ice for 5 min. Samples were fractionated at 15 kV for 6 s and at 15 kV for 33 min at 66°C.

### Methylation Sensitive Genotyping by Sequencing

We performed the methylation-sensitive modification of the genotype by sequencing (GBS) technique (Poland et al., 2012) as described by Xia et al. (2014). In brief, a two-enzyme approach was used to generate restriction products. In this experiment, only one enzyme combination was used *(Msp*I with *EcoR*I). The selected enzyme combination was based on the results obtained using the MSAP approach. Two hundred nanogram of genomic DNA from each of the 86 selected samples [comprising three replicate per tissue/variety and growing condition (i.e., *in vitro* or field) see Supplementary Table S1] were used in a reaction volume of 20 μl containing 2 μl of NEB Smart cut buffer, 8 U of HF-*EcoR*I (High-Fidelity) and 8 U of *Msp*I (New England BioLabs Inc., Ipswich, MA, USA). Reactions were prepared in a 96 well plate containing 87 reactions (86 DNA samples plus one Negative control water sample) and conducted on a BioRad 100 thermocycler at 37°C for 2 h and then 65°C for 20 min for enzyme inactivation. A set of 96 barcoded adapters with an *Msp*I overhang and a common Y adapter with an *EcoR*I overhang were designed for the ligation reaction using barcode script made by Thomas P. van Gurp^1^. Adapters were annealed prior to ligation as described by Poland et al. (2012). A full list of adapters for *Msp*I (with corresponding barcodes and cassava samples) and *EcoR*I is listed in Supplementary Table S1. The ligation reaction (40 μl) was carried out on the same PCR plate adding to the restriction products T4 Ligase (200 U) and T4 Ligase buffer (NEB T4 DNA Ligase #M0202), 0.1 pmol of the respective barcoded *Msp*I adapter and 15 pmol of the common Y-adapter. Ligation was completed at 22°C for 2 h followed by an enzyme inactivation step of 20 min at 65°C. Five micro liter from each ligation reaction were pooled into a single tube and then divided into two equal volumes for column clean-up using PureLink^®^ PCR Purification Kit (Life Technologies) according to manufacturer’s instructions. Samples were re-suspended in 60 μl of nanopure water. Both clean-ups were then combined and divided again into eight samples for PCR amplification. Each 25 μl PCR consisted of 10 μl of DNA digested/ligated library), 5 μl of 5x NEB MasterMix, 2 μl of 10 uM Forward and Reverse primers at 10 uM (Supplementary Table S1). Reactions were performed in a BioRad T100 thermocycler for eight cycles consisting of 95°C (30 s), 62°C (30 s), 68 °C (30 s). All eight PCR products were pooled and then purified first using a PureLink^®^ PCR Purification Kit (Life Technologies) according to manufacturer’s instructions (resuspended in 30 μl). Excess adaptor was finally removed using Ampure XP magnetic beads (Beckman) by mixing 30 μl of the pooled PCRs with 22.5 μl of beads. Captured fragments were eluted in 30 μl of water. Next, 125 bp paired-end sequencing was performed in one Illumina HiSeq 2000 v4lane (Illumina Inc., San Diego, CA, USA) by QBI Centre for Brain Genomics.

### Statistical Analysis

#### Analysis of Genetic/Epigenetic Variability using MSAP

The MSAP technique uses MspI or HpaII as isoschizomers; both can cleave the motif CCGG in the absence of methylation. MspI can also cleave hemi-methylated dsDNA (mC in one DNA strand only) or fully methylated DNA sequences where the internal cytosine is methylated C^m^CGG. However, it cannot digest hemi-methylated and fully methylated at the external cytosine site, viz: mCCGG and mCmCGG motifs (Walder et al., 1983; Reyna-Lopez et al., 1997). In contrast, HpaII is more sensitive to methylation but can cleave hemimethylated DNA at the external cytosine position (mCCGG; Mann and Smith, 1977; Reyna-Lopez et al., 1997). Direct comparison of MspI profiles with those generated by the more methylation-sensitive HpaII therefore does not provide a definitive contrast between genetic variation and that attributable to changes in methylation (Fulneček and Kovařík, 2014). For these reasons, simple comparisons were made between profiles generated from various tissues of plants grown in the two settings (micropropagation and field cuttings) under the reasonable assumption that consistent differences will arise from differential methylation (driven by RdDM) rather than by repeated chance mutations.

MSAP profiles were visualized using GeneMapper Software v4 (Applied Biosystems, Foster City, CA, USA). Two matrices containing allelic information were generated. First, a qualitative analysis was carried out in which epiloci were scored as “present” (1) or “absent” (0) to form a presence/absence binary matrix. In this case, the selection of MSAP fragments was limited to allelic sizes between 100 and 580 bp to reduce the potential impact of size homoplasy (Caballero et al., 2008). Profile polymorphisms between DNA samples from the same cassava variety but extracted from different tissues (young leaves, newly mature leaves, and primary root tips) were retained as inter-tissue methylation differences. Polymorphisms between DNA samples from *in vitro* culture plants and from field grown plants were considered as *in vitro* culture induced methylation differences. Second, a matrix containing peak heights of fragments with allelic size between 50 and 550 bp was created for quantitative analysis (Rodríguez López et al., 2012). In both cases, different levels of hierarchy were generated to group the samples. Samples were first grouped according to cassava variety. Then, samples were divided into field grown and *in vitro* grown. Finally, samples were separated into the three different tissues of origin (young leave, mature leave, and roots).

For the analysis of the MSAP qualitative data, GenAlex v6.4 software (Peakall and Smouse, 2006) was used to infer pairwise epigenetic PhiPT distances (estimation of genetic/epigenetic distances) between different cassava samples. Analysis of molecular variance (AMOVA) was then performed using the same software to test the significance of the estimated PhiPT between tissues (Michalakis and Excoffier, 1996). An allele frequency table was generated using GenAlex 6.4 to find *in vitro*/field specific qualitative markers for each cultivar and for all cultivars. Finally, the visualization of the patterns of tissue epigenetic variations in this study was done by constructing a Principal Coordinates Analysis (PCoA).

For each variety, the peak height intensities of the epiloci generated using MSAP were compared between field grown and *in vitro* tissue samples as well as compared between samples from different tissue origins within field and *in vitro* groups. Preceding comparative analysis the data was filtered by removing epicloci containing excessively low peak height intensities across the complete set of samples. From this reduced set of epiloci the peak height libraries were normalized using the model based weighted trimmed mean method derived in Robinson and Oshlack (2010). For each pair of tissue groups being investigated, the normalized peak heights were extracted and compared using the approach described in Robinson and Smyth (2007, 2008). This approach initially assumes the normalized peak heights are distributed as a negative binomial with a common dispersion calculated across the complete set of epiloci for the two groups. From this, individual epiloci dispersions were calculated using the empirical Bayes methods of Robinson and Smyth (2007). An exact statistical test was then conducted for each epiloci to determine differences in peak heights between the two groups (Robinson and Smyth, 2008). The *p*-values obtained from these tests were then appropriately adjusted for multiple comparisons using the false discovery rate (FDR) method of Benjamini and Hochberg (1995). All analyses were performed using the differential expression analysis R package edgeR (Robinson et al., 2010) available in the R statistical computing environment (R Development Core Team Foundation, 2015).

#### Analysis of Genetic/Epigenetic Variability using GBS Data

For the processing of Illumina HiSeq 2000 v4 data, the sequences from the unfiltered fastq Illumina output were separated into samples using the barcode sequence and trimmed to 64 bp using the software TASSEL (sourceforge.net/projects/tassel/). Only sequences with one of the exact used barcodes followed by the expected sequence of three nucleotides remaining from an *Msp*I cut-site (5′-CGG-3′) were retained for analysis. Sequences present in the negative water control were also removed from the analysis. Finally only sequences present in three or more different samples were kept for analysis. A matrix of sequence abundance of was then generated for further analysis.

Using the differential expression analysis procedure outlined in Section “Analysis of Genetic/Epigenetic Variability Using MSAP,” the sequence abundances were compared between field grown and *in vitro* tissue samples and also compared between samples from different tissue origins within field and *in vitro* groups. Sequences presenting significantly different number of reads between all *in vitro* and all field grown samples for each variety were isolated. Finally, only those sequences that presented the same variation (increase or reduction of number of reads in all varieties when comparing *in vitro* against field grown samples) in at least four of the five studied varieties were considered micropropagation induced markers. Due to the extremely low probability of a mutational event leading to the generation of these markers happening in all plants from all varieties during culture we can consider that such markers are differentially methylated regions (DMRs) induced by the micropropagation procedure. Detected DMRs were then selected for blast analysis against the cassava (BLASTN, nucleotide query to cassava nucleotide database genome blast tool in Phytozome. Top hits indicating differential methylation of a genic region were sought by comparing exons, introns, and flanking sequences (5 kb upstream of the Transcription Start Site and 5 kb downstream of the Transcription Termination Site).

## Results

### Analysis of Genetic/Epigenetic Variability using MSAP

#### Estimation of Genetic/Epigenetic Differences Based on Qualitative Analysis

**FIGURE 1:**
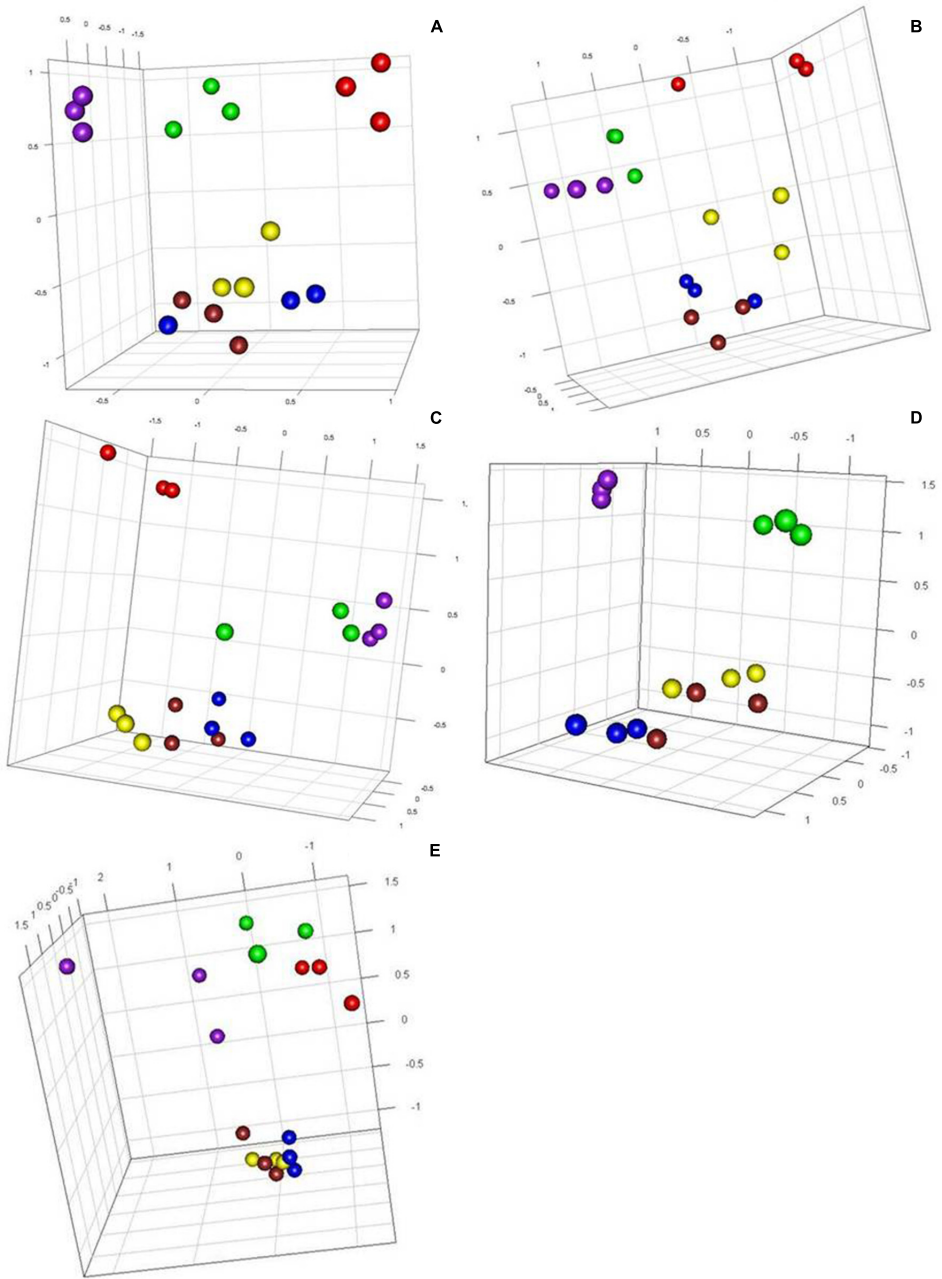
**Principal component analysis showing tissue separation in five cassava varieties using generated epigenetic profiles**. Effects of tissue culture and field environment on epigenetic variation. Principal coordinate diagrams based on the Euclidean analysis of methylation-sensitive amplified polymorphism (MSAP) distances obtained from three different tissues of Kiroba **(A)** and Mzungu **(B)**, Kizimbani **(C)**, Mfaransa **(D)** and Kibandameno **(E)** varieties (*in vitro* and field cuttings) using primer combination *Hpa*II2.2/*EcoR*I5. Green, field young leaves; Red, field mature leaves; Purple, field roots; Yellow, *in vitro* young leaves; Blue, *in vitro* mature leaves; and Brown, *in vitro* roots.

Methylation-sensitive amplified polymorphism profiles generated a total of 164 loci (13 unique to *Hpa*II, 22 unique to *Msp*I, and 129 common to both enzymes) for the 86 samples of five cassava cultivars used in this study. PCoA analysis created from a simple presence/absence similarity matrix of the combined MSAP profiles revealed clear separation between *in vitro* propagated cultivars and their field counterparts for all cultivars (**Figure 1**). Calculated genetic/epigenetic distances between field and *in vitro* samples were significant for all cultivars (**Table 2**). In general, calculated distances between *in vitro* and field samples where higher for samples restricted using *Msp*I (**Table 2**). All pairwise PhiPT between *in vitro*-grown samples and those grown in the field tissues were significant for all varieties using both *Hpa*II and *Msp*I (**Table 2**). In general, genetic/epigenetic distances were higher between tissues recovered from plants grown in the field than between those taken from *in vitro-*grown plants (**Table 3**). The reduced divergence between tissues taken from *in vitro*-grown material was also evident from the PCoA analysis, with samples from different tissues of *in vitro* propagated plants occupying less eigen space than those of the same tissues obtained from field grown plants (**Figure 1**). However, the level of variability observed within tissue types did not differ significantly between *in vitro-*grown and field-grown material.

**Table 2:** Summary of calculated genetic/epigenetic distance (PhiPT) between *in vitro-* and field-grown samples.

	*Hpa*II		*Msp*I	
	PhiPT	*p*-value	PhiPT	*p*-value
Mzungu	0.159	0.006	0.188	0.001
Kiroba	0.130	0.002	0.310	0.001
Kibandameno	0.273	0.001	0.374	0.001
Mfaransa	0.577	0.001	0.541	0.002
Kizimbani	0.160	0.003	0.243	0.001

*Pairwise genetic/epigenetic distance between samples grown in vitro and those grown in the field from each variety were calculated using MSAP profiles obtained by restricting gDNA from five cultivars of cassava with EcoRI and HpaII or MspI using GenAlex 6.5.1 AMOVA. p-values were calculated based on 1000 permutations*.

**Table 3:** Effect of *in vitro* culture on epigenetic differentiation between tissues.

Tissues	*Hpa*II			*Msp*I	
	Cultivar	Field	*In vitro*	Field	*In vitro*
Ylv-Mlv	Kibandameno	0.208	0.070	0.237	0.000
	Mzungu	0.000	0.042	0.239	0.192
	Mfaransa	–	0.112	–	0.209
	Kizimbani	0.171	0.000	0.114	0.071
	Kiroba	0.259	0.178	0.260	0.118
	**Average**	**0.1595**	**0.0804**	**0.2125**	**0.118**
Ylv-Rt	Kibandameno	0.447	0.296	0.308	0.197
	Mzungu	0.000	0.162	0.461	0.178
	Mfaransa	0.401	0.260	0.538	0.148
	Kizimbani	0.142	0.272	0.654	0.286
	Kiroba	0.264	0.098	0.250	0.090
	**Average**	**0.2508**	**0.2176**	**0.4422**	**0.1798**
Mlv-Rt	Kibandameno	0.196	0.301	0.049	0.362
	Mzungu	0.108	0.344	0.459	0.386
	Mfaransa	–	0.408	–	0.375
	Kizimbani	0.263	0.139	0.550	0.250
	Kiroba	0.357	0.201	0.344	0.212
	**Average**	**0.231**	**0.2786**	**0.3505**	**0.317**

*Calculated pairwise PhiPT values (epigenetic distances) between tissues from field cuttings and in vitro propagated lines. Distances were calculated using GenAlex 6.5.1 AMOVA on MSAP profiles obtained by restricting genomic DNA with MspI and HpaII. Ylv, young leaves, Mlv, mature leaves, Rt, roots*.

We further analyzed the differences existing between each tissue derived from the field grown plants and all samples from plants grown *in vitro.* The aim here was to investigate which of the *in vitro*-grown tissues generated MSAP profiles were most similar to the field samples. Distance estimates were significant for all pairs, but it was consistently smaller between young leaves from field grown plants and bulked *in vitro* tissues (**Table 4**).

**Table 4:** Epigenetic distance between field cutting tissues and all tissues from *in vitro* conditions.

	Kizimbani	Mzungu	Kiroba	Mfaransa	Kibandameno
Young lv	0.236^∗^(0.007)	0.276^∗^(0.004)	0.308^∗^(0.006)	0.559^∗^(0.026)	0.502^∗^(0.007)
Mature lv	0.350 (0.001)	0.286 (0.003)	0.423 (0.007)	Δλ	0.521 (0.004)
Root	0.528 (0.005)	0.398 (0.006)	0.389 (0.007)	0.655 (0.006)	0.576 (0.007)

*Calculated pairwise tissue PhiPT values (epigenetic distances) obtained from MSAP profiles obtained by restricting genomic DNA from ten cultivars of cassava with MspI using GenAlex 6.5.1 AMOVA. The values show the distance between individual tissues of field cutting lines from bulks of all tissues of in vitro propagated lines. Young lv: distance between field derived young leaves and bulked in vitro tissues, Mature lv: distance between field derived mature leaves and bulked in vitro tissues, and Root: distance between field derived root and bulked in vitro tissues. Probability values based on 9999 permutations are shown in parenthesis. Asterisks indicate lower PhiPT values*.

#### Estimation of Genetic/Epigenetic Differences Based on Quantitative Analysis

We selected 106 markers for quantitative analysis of MSAP profiles based on peak height data. In general, both enzymes, yielded more markers separating between tissues from field-grown plants than those taken from *in vitro* material (62 vs. 44 for *Msp*I and 44 vs. 42 for *Hpa*II; **Table 5**; For a list of all fragments and their levels of significance see Supplementary Table S2 for *Msp*I and Supplementary Table S3 for *Hpa*II). However the number and scale of these differences varied between cultivars.

**Table 5:** Number of significantly different quantitative epimarkers across all cultivars.

Cultivar	*Msp*I			*Hpa*II		
	Field vs. *in vitro*	Tissues (*in vitro*)	Tissues (Field)	Field vs. *in vitro*	Tissues (*in vitro*)	Tissues (Field)
Mfaransa	6	10	7	6	9	2
Mzungu	0	9	19	1	9	7
Kizimbani	3	1	27	2	7	7
Kiroba	8	13	8	3	12	11
Kibandameno	6	11	1	7	5	17

*Quantitative markers were generated from MSAP profiles peak heights obtained by restricting genomic DNA from five cultivars of cassava (in vitro and in the field) with MspI and HpaII. Column Field vs. in vitro shows the number of significantly different (p > 0.005) epimarkers between samples of the same cultivar grown in vitro or in the field. Columns Tissues (in vitro) and Tissues (Field) show the number of significantly different (p > 0.005) epimarkers between tissues grown either in the field or in vitro.*

**FIGURE 2:**
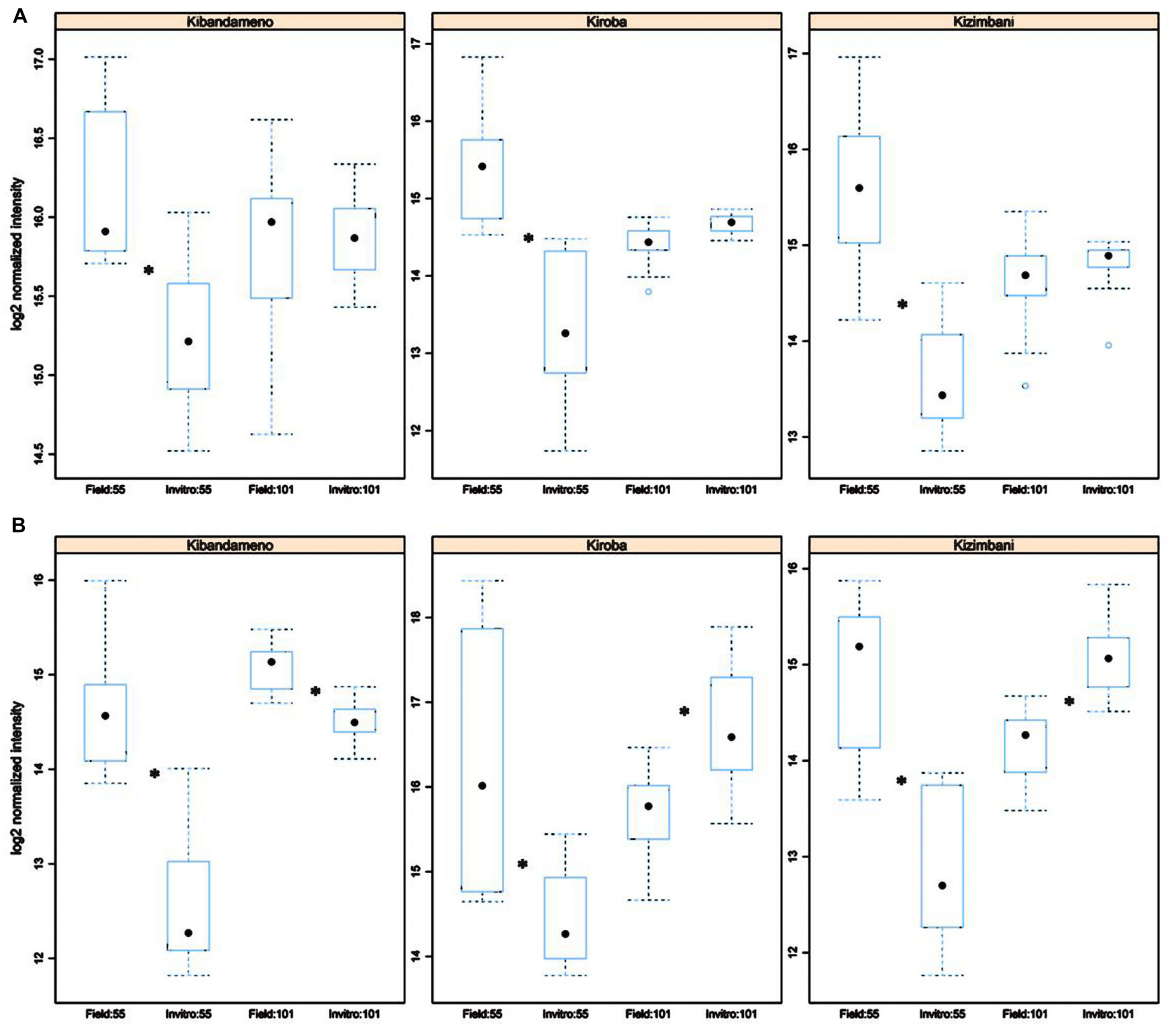
**Differential peak intensity of informative epiloci (55 and 101 bp) between *in vitro* tissues and field tissues for three cassava cultivars (Kibandameno, Kiroba, Kizimbani)**. Peak intensities were obtained from MSAP profiles generated restricting genomic DNA from three different tissues (i.e., young leaf, mature leaf, and roots) in three plants grown either *in vitro* or in the field with *Msp*I **(A)** and *Hpa*II **(B)** and amplifying using primer combination *Hpa*II2.2/*EcoR*I5. Box plots show the average normalized intensity scores for a 55 and a 101 bp MSAP fragment selected using. Asterisk indicates *p* < 0.005.

A total of 14 and 15 markers for *Msp*I and *Hpa*I respectively were found to be significantly different (*p* < 0.005) between all *in vitro*- and field-derived material. Two of these markers generated using *Hpa*II, epiloci 55 and 101 bp, were able to diagnose *in vitro* from field samples of three varieties, Kiroba, Kibandameno, and Kizimbani (**Figure 2**). The epilocus 55 bp generated using *Msp*I was also significantly different between propagation systems for the same varieties but not epilocus 101 bp (**Figure 2**).

### Analysis of Epigenetic Variability using GBS Data

In total, we generated 236,624,193 raw reads from the HiSeq 2000 v4 lane, of which 71,723,843 (32.3%) passed quality filter and contained the expected exact matches to sequences of the barcode adapter, *Msp*I restriction product site and the *EcoR*I adapter, and which appeared in at least three biological samples but were absent from the negative (water) control. On average, 754,980 high quality reads were produced per DNA sample. Collectively, this included 357,271 unique sequence tags across all samples. The number of these markers that differed significantly in abundance between *in vitro*-grown and field-grown samples varied considerably between varieties: 3,298 for Kiroba; 25,683 for Kizimbani; 2,029 for Mfaransa; 34,098 for Mzungu and 17,702 for Kibandameno. Most of these sequences were more abundant in the field-grown samples (**Table 6**) and the overwhelming majority was variety-specific responses. We next sought to identify candidate generic epimarks that differentiate between propagation systems across all varieties. When the most stringent filter for differential abundance was applied (i.e., reads with an FDR lower than 0.05; the phase of differential abundance being conserved across all genotypes and tissues, and the absence of variety-specific SNPs) just 105 (0.03%) of unique differential sequences featured in the profiles of all varieties and showed a common pattern of phasing (Supplementary Table S4). There was also a marked difference in the phase of these marks, with just four tags being more abundant among *in vitro*-grown samples compared with 101 that were more numerous in the field-grown plants.

**Table 6:** Number of significantly different msGBS sequences between *in vitro* and field grown samples.

Cultivar	*In vitro*	Field	Total
Mfaransa	185	1844	2029
Mzungu	16021	17888	34098
Kizimbani	2245	23438	25683
Kiroba	1918	1380	3298
Kibandameno	5068	12634	17702

*“In vitro” and “Field” columns indicate the number of sequences with a higher number of reads from samples grown under each condition*.

When compared against the cassava nucleotide database genome using the blast tool in Phytozome^2^, 102 differentially methylated sequences generated one or multiple hits against the cassava genome. Eighty nine top hits were associated to a gene (i.e., mapped within a window of 5 kb from the gene). BLAST results indicate that the homologs of these genes are involved in many processes, including cell differentiation, plant development, sugar metabolism, nucleic acid methylation, stress response, photosynthesis, signaling and transposon activation (Supplementary Table S5). Of the 89 differentially methylated genes 45 have been previously mentioned in the literature as having homologous genes that are: regulated by DNA methylation (14) or other epigenetic mechanisms (10), methylated or differentially methylated under different growing conditions (4), implicated in the regulation of DNA methylation (9) or other epigenetic mechanisms (1) and DNA binding proteins affected by methylation of their target sequences (3).

## Discussion

Micropropagation via nodal cuttings relies on the regeneration of pre-existing meristems and so is widely considered to be analogous to field cuttings because they do not pass through a state of disorganized (dedifferentiated) tissue state (Dale and McPartlan, 1992). Nevertheless, ramets recovered from nodal micropropagation can still exhibit signs of increased morphological variability (somaclonal variation) when compared to those recovered from field cuttings (Debnath, 2005). Us-Camas et al. (2014) suggested that such observations might be explained by the stressful environment experienced by *in vitro*-grown plants (i.e., high relative humidity, low ventilation rate, high concentrations of sugars and plant growth regulators, and low light availability). Under these conditions, cultured plants cells are also forced to change their molecular make ups in order to generate different cell types. Cell division to generate tissues and organs require a precise coordination of genetic and epigenetic processes (Miguel and Marum, 2011; Smulders and de Klerk, 2011). For micropropagation systems that rely on dedifferentiation and *de novo* organogenesis, *in vitro* culture can often yield occasional regenerants that are phenotypically off-type (e.g., Lakshmanan and Taji, 2000; Rout et al., 2000; Da Silva et al., 2015). In contrast, those recovered from meristem micropropagation are widely reported to remain more faithful to the phenotype of the parental plant in range of species (e.g., Villordon and LaBonte, 1996) including cassava (e.g., Santana et al., 2009). There is nevertheless a large body of evidence indicating that changed growing conditions often induces moderations in global methylation patterns in culture (for review see Pastor et al., 2013) and this leads to the plausible expectation of epigenetic divergence between plants cloned by meristem micropropagation and field cuttings. Evidence supporting this assertion came from a study by Baranek et al. (2010), who used MSAP profiles to compare daughter plants recovered from field cuttings and micropropagated nodal segments of two grape vine varieties. The authors found consistent differences between the two systems in their clustering on resultant dendrogams. However, the work failed to further characterize the variation in terms of tissue type or to provide sequence identity for the differential epimarks. Characterizing such epigenetic differences may prove useful not only for the mere detection of putative somaclonal variants (Causevic et al., 2006) but for use in epiallele discovery, and as a tool for directed crop epigenetic improvement. In this study we combine MSAP and msGBS (Xia et al., 2014) to survey for *C*-methylation perturbations associated with the micropropation of elite clones of cassava (*M. esculenta*).

### Analysis of Genetic/Epigenetic Variability using MSAP

Both quantitative (**Figure 1**) and qualitative analysis (**Table 5**) of MSAP generated profiles showed clear separation of all tissues in all five varieties studied. Higher levels of diversity and divergence were observed when using *Msp*I than *Hpa*II (**Table 2** and **4**). Care should be exercised before tentatively assigning this variability as likely to have arisen through genetic or epigenetic causes. Moreover, polymorphic markers between propagation systems that appear in the profiles generated of both isoschizomer restriction enzymes (*Hpa*II and *Msp*I; **Figure 2**) could be caused by either a genetic or an epigenetic change. Conversely, a polymorphic marker detected by only one of the enzymes can only be epigenetic in nature (Pérez-Figueroa, 2013). Application of this reasoning implies that variation at the propagation system diagnostic 55 epilocus could be explained by both genetic and epigenetic changes whereas that of epilocus 101 was due to differential methylation arising from the tissue culture conditions (**Figure 2**). However, since the chance of a genetic mutation occurring at exactly the same location on more than one occasion is extremely low (Rodríguez López et al., 2010b) combined with the fact that these two markers polymorphic were found in three different cultivars implies that both markers probably have an epigenetic origin rather than one caused by genetic mutation.

The variability in MSAP profiles seen between DNA extracted from the same tissue type was both modest and consistent, regardless of the propagation system used to produce the plants (**Figure 1**). This finding suggests that these DNA methylation changes induced by micropropagation are not random, as would be expected for genetic somaclonal variation (Bairu et al., 2011) and so more likely to be associated with methylation events associated with cell and tissue differentiation. Circumstantial evidence in support of this inference can be taken from the PhiPT distance estimate, which showed that samples from *in vitro* nodal micropropagation ramets were always (epigenetically) closer to young leaves of their field counterparts (**Table 4**).

Genetic variation induced during *in vitro* nodal micropropagation cannot be ruled out in this study. However, our results suggest that the majority, if not all, the variability detected using MSAPs is epigenetic in nature. This is supported by the lack of higher levels of variation between micropropagated samples than in field grown samples and the fact that the observed variability seems to be conserved between different plants and between different varieties (**Figure 1**) and also by previous studies that show that micropropagated plants using this approach present high levels of genetic stability (Lata et al., 2010) but measurable levels of epigenetic variability (Baranek et al., 2010).

Our MSAP results suggest that (1) *in vitro* nodal micropropagation introduces *de novo* variability in the global methylation patterns; (2) micropropagation induced epigenetic variability does not seem to be random.

### Analysis of Epigenetic Variability using GBS Data

Most studies of the epigenetic basis of somaclonal variation have used MSAPs to characterize culture-induced epigenetic variation. This technique is reliable and does not require previous knowledge of the studied organism. Conversely, it presents the disadvantage that the generated markers are anonymous. It is possible to isolate and sequence the differential markers (Massicotte et al., 2011), although the process can be cumbersome, expensive and time-consuming (Schrey et al., 2013), especially when many markers and samples are involved. The use of Next-generation sequencing can significantly reduce the cost of epiallele sequence characterization. The recent development of GBS (Elshire et al., 2011; Poland et al., 2012) and its methylation-sensitive version (ms-GBS; Xia et al., 2014) has allowed for a simple, time and cost effective system for the sequencing of multiple DMRs in non-model organisms.

Our study uncovered 105 unique sequences (Supplementary Table S4; 0.03% of those generated) with different levels of methylation between propagation systems. Although total sequence reads were similar between systems, the vast majority of differential tags (101/105) were more abundant among ramets recovered from field cuttings, suggesting again lower global levels of methylation in field grown plants. This is in contradiction with previous evidence suggesting that in vitro culture is related to low DNA methylation (Valledor et al., 2007). However, deciphering global hyper/hypomethylation from restriction products is not a reliable approach (Fulneček and Kovařík, 2014). What is more, other studies have shown that methylation levels during in vitro propagation are related to the donor tissue (Fang et al., 2009; Wang et al., 2012), to the length of the culture (Diaz-Sala et al., 1995; Rodríguez López et al., 2010a,b), and the media components (LoSchiavo et al., 1989; Arnholdt-Schmitt, 1993).

BLAST analysis against the cassava genome of the micropropagation induced DMRs generated in this study yielded significant hits for 102 sequences of which the 89 top hits were each associated to a gene (i.e., mapped within a window of 5 kb from the gene; Supplementary Table S5). BLAST results indicate that the homologs of these fragments are involved in many processes, including cell differentiation, plant development, sugar metabolism, nucleic acid methylation, stress response, photosynthesis, cell wall modifications, signaling and transposon activation (Supplementary Table S5). However, it is important to remember that the mere presence of differential methylation in or around a gene is not sufficient evidence to infer that expression of the gene is actually regulated by methylation. There are nevertheless enticing hints to suggest that these candidate methylation markers for propagation system may indeed also play a role in metabolic divergence between field cuttings and meristem micropropagated plants.

A series of studies have implicated DNA methylation in the regulation of genes controlling pathways in plant developmental progression or tissue differentiation (Messeguer et al., 1991), during embryogenesis, seed formation (Xiao et al., 2006), apical dominance regulation, flowering, and floral and leaf formation (Finnegan et al., 1996). Several differentially abundant loci identified in the present study showed high sequence homology to loci in cassava that have been previously implicated in cell differentiation and development: CWF19, XPMC2, EXO70, TAP42-like, Sterile alpha motif (SAM) domain-containing protein, AP3M, Enhancer of polycomb-like transcription factor protein, cassava protein containing a transcription factor UCC1, GFS9, GT-2, EMB71, and ARF2 (Supplementary Table S5). Even if it were shown that the changes in methylation among loci identified are causally linked to changes in gene expression, further work would still be required to establish whether such changes are sufficient to cause a biological meaningful change in cell metabolism and phenotype. Once again, however, there are some grounds to reason that at least some loci might.

A number of studies have shown that DNA methylation plays a central role in gene expression and plant development under stress (for extensive reviews see Chinnusamy and Zhu, 2009; Kinoshita and Seki, 2014). Not surprisingly perhaps, abiotic stresses like those encountered under *in vitro* culture conditions have been found to impose an effect on DNA methylation and have been correlated with subsequent organogenesis (Us-Camas et al., 2014). However, the comparative paucity of marks that appear at higher abundance in meristem culture (just 4 of the 105 generic marks; Supplementary Table S4) suggests that changes of this type lay in the minority. Explanation is therefore required for the far more commonly encountered appearance of marks among the field cutting samples only. Perhaps the most plausible hypothesis for this divergence lay in the more variable living environment experienced by field cutting plants when compared with the more homogeneous environment in culture. Field-grown plants are continuously exposed to pathogens throughout their lifetime and their DNA epigenetic patterns become altered by infection (Alvarez et al., 2010; De-La-Peña et al., 2012). It is therefore, not difficult to presume that *in vitro* grown plants will not experience the same pathogen or abiotic stress-induced DNA methylation marks as will those grown exposed to pathogens in the field. In our study, 13 of the 105 differentially methylated loci mapped to locations of the cassava genome associated to known stress response genes [i.e., Calcium-dependent lipid-binding (CaLB domain) family protein, Plastocyanin-like domain, Leucine-Rich Repeat Receptor-Like Protein Kinase (LRR-RK), Disease resistance protein (TIR-NBS-LRR class) family, ATBCB, ATHCHIB, ATTTM2, MLP-Like Protein 28, ATMGL, ATBZIP1, XTH1, Peroxidase superfamily protein, ATATG18F and HT1; Supplementary Table S5]. Plants have evolved two different strategies involving LRR proteins to perceive microbial pathogens. LRR-RKs are transmembrane host-encoded pattern-recognition receptors that directly recognize pathogens while NBS-LRR indirectly recognize pathogen effectors by sensing their effects on plant target proteins (Yu et al., 2013). The latest has been shown to be regulated by DNA methylation (Yu et al., 2013). Previous studies have shown that overexpression of NBS-LRRs induces a severe drop in fitness (Tao et al., 2000). Our results show several of the 105 candidate loci associated to both types of LRRs, so it would be tempting to speculate that the agronomic performance of micropropagated plants could be potentially affected if the observed changes on DNA methylation led to the overexpression of such genes. Calcium lipid-binding domain (CaLB domain) proteins are repressors of abiotic stress response in plants (de Silva et al., 2011) and have been shown to be regulated by environmental conditions through DNA methylation (Dubin et al., 2015). Curiously, our results showed one differentially abundant sequence matched to the xyloglucan endotransglucosylase/hydrolase1 (XTH1) gene. Previous studies have shown that XTHs have a function on cell wall modifications and that changes on their DNA methylation levels are associated to colonization of potato plants by beneficial bacterial endophytes (Da et al., 2012).

In all, 45 of the 89 differentially abundant sequences matched to genes that have been previously reported to be: regulated by DNA methylation (14) or other epigenetic mechanisms (10), methylated or differentially methylated under different growing conditions (4), associated to the regulation of DNA methylation (9) or other epigenetic mechanisms (1) and DNA binding proteins affected by methylation of their target sequences (3) (For references see Supplementary Table S5). Which (if any) of these genes is playing a role in a possible divergence in cell metabolism and phenotype between plants replicated by meristem-propagation and field cutting warrants further attention. Looking further ahead, identifying the developmentally important genes whose expression is sensitive to culture growth conditions may ultimately allow for the development of new culture regimes that yield regenerants with the lowest possible incidence of off-types. In the shorter term, however, the provision of methylation marks that consistently diverge in abundance between plants propagated by meristem culture and those recovered by field cuttings could have utility in the optimization of *in vitro* meristem propagation protocols and also in the diagnosis of the origin of clonal stocks.

## Conflict of Interest Statement

The authors declare that the research was conducted in the absence of any commercial or financial relationships that could be construed as a potential conflict of interest.

## Footnotes

1 www.deenabio.com/gbs-adapters.

2 http://www.phytozome.net
