# Supplementary material for: Meristem micropropagation of cassava (Manihot esculenta) evokes genome-wide changes in DNA methylation
Source: Front Plant Sci. 2015 Aug 13;6:590. doi: 10.3389/fpls.2015.00590 (PMC4534864; doi:10.3389/fpls.2015.00590)
Supplement: Supplementary file 6 [file Supplementary_references.PDF]

Meristem micropropagation of cassava (*Manihot esculenta*) evokes genome-wide changes in DNA methylation

Shedrack R. Kitimu[1,5], Julian Taylor[2], Timothy J March[3], Fred Tairo[4], Mike J Wilkinson[1] and Carlos Marcelino Rodríguez López[1]*

[1]Plant Research Centre, School of Agriculture Food and Wine, Faculty of Sciences, University of Adelaide, Waite Campus, PMB1 Glen Osmond. *SA 5064. Australia.*

[2]Biometry Hub, Waite Building, School of Agriculture Food and Wine, Faculty of Sciences, University of Adelaide, Waite Campus, PMB1 Glen Osmond. *SA 5064. Australia.*

[3]Waite Building, School of Agriculture Food and Wine, Faculty of Sciences, University of Adelaide, Waite Campus, PMB1 Glen Osmond. *SA 5064. Australia.*

[4]Mikocheni Agricultural Research Institute, Dar es Salaam, Tanzania

[5] Current address: Sokoine University of Agriculture, P.O BOX 3000, Morogoro Tanzania

**Correspondent author*

Correspondence:

Dr. Carlos Marcelino Rodríguez López

University of Adelaide

Plant Research Centre

School of Agriculture, Food and Wine

Waite Campus, Adelaide, SA, 5064, Australia.

carlos.rodriguezlopez@adelaide.edu.au

Supplementary references

Bittencourt, D., Lee, B.H., Gao, L., Gerke, D.S., Stallcup, M.R. (2014). Role of distinct surfaces of the G9a ankyrin repeat domain in histone and DNA methylation during embryonic stem cell self-renewal and differentiation. *Epigenetics & Chromatin*. **7**, 27. doi:10.1186/1756-8935-7-27

Derreumaux, S., Chaoui, M.,Tevanian, G., Serge Fermandjian, S.,(2001). Impact of CpG methylation on structure, dynamics and solvation of cAMP DNA responsive element. *Nucl. Acids Res*. 29 (11), 23142326.doi: 10.1093/nar/29.11.2314

Escalante-Alcalde, D., Recillas-Targa, F., Hernández-García, D., Castro-Obregón, S., Terao, M., Garattini, E., Covarrubias, L. (1996). Retinoic acid and methylation cis-regulatory elements control the mouse tissue non-specific alkaline phosphatase gene expression. *Mech Dev*. 57(1):21-32.

Friso, S., Choi, S.-W., Girelli, D., Mason, J.B., Dolnikowski, G.G., Bagley, P.J., Olivieri, O., Jacques, P.F., Rosenberg, I.H., Corrocher, R., Selhub, J. (2002). A common mutation in the 5,10-methylenetetrahydrofolate reductase gene affects genomic DNA methylation through an interaction with folate status. *Proc. Natl. Acad. Sci.* USA. 9(8), 5606–5611. doi_10.1073_pnas.062066299

Jeon, J., Choi, J., Lee, G.-W., Park, S.-Y., Huh, A., Dean, R.A., Lee, Y.-H. (2015) Genome-wide profiling of DNA methylation provides insights into epigenetic regulation of fungal development in a plant pathogenic fungus, *Magnaporthe oryzae*. *Sci. Rep*. 5, 8567; DOI:10.1038/srep08567

Kimatu, J.N., Diarso, M., Song, C., Agboola, R.S., Pang, J., Qi, X., Liu, B. (2011). DNA cytosine methylation alterations associated with aluminium toxicity and low pH in *Sorghum*

bicolor. *African Journal of Agricultural Research*. 6(19), 4579-4593. DOI: 10.5897/AJAR11.954

Li, R.-Q., Huang, J.-Z., Zhao, H.-J., Fu, H.-W., Li, Y.-F., Liu, G.-Z., Shu, Q.-Y. (2014). A down-regulated epi-allele of the *genomes uncoupled 4* gene generates a *xantha* marker trait in rice. *Theor Appl Genet*. 127, 2491–2501. DOI 10.1007/s00122-014-2393-9

Lisanti, S., (2013). Changes In Dna Methylation Patterns In Mammals With Senescence, Ageing And Energy Restriction. Thesis submitted for the degree of Doctor of Philosophy. New Castle University, UK.

Lisch, D. (2009). Epigenetic Regulation of Transposable Elements in Plants. *Annu. Rev. Plant Biol*. 60, 43–66

Lister, R., O'Malley, R.C., Tonti-Filippini, J., Gregory, B.D., Berry, C.C., Millar, A.H., Ecker, J.R. (2008). Highly integrated single-base resolution maps of the epigenome in Arabidopsis. *Cell*. 133(3):523-36.

Luo, X., Gao, Z., Shi, T., Cheng, Z., Zhang, Z., Ni, Z. (2013). Identification of miRNAs and Their Target Genes in Peach (*Prunus persica* L.) Using High-Throughput Sequencing and Degradome Analysis. *PLoS ONE*. 8(11), e79090. doi:10.1371/journal.pone.0079090

Shan, X., Wang, X., Yang, G., Wu, Y., Su, S., Li, S., Liu, H., Yuan, Y. (2013). Analysis of the DNA Methylation of Maize (*Zea mays* L.) in Response to Cold Stress Based on Methylation-sensitive Amplified Polymorphisms. *J. Plant Biol*. 56, 32-38. DOI 10.1007/s12374-012-0251-3

Suganuma, T., Pattenden, S.G., Workman, J.L. (2008). Diverse functions of WD40 repeat proteins in histone recognition. *Genes & Development*. 22, 1265–1268

Sun, W., Iijima, T., Kano, J., Kobayashi, H., Li, D., Morishita, Y., Okubo, C., Anami, Y., Noguchi, M. (2008). Frequent aberrant methylation of the promoter region of sterile a motif domain 14 in pulmonary adenocarcinoma. *Can Sci*. 99(1), 2177–2184. doi: 10.1111/j.1349-7006.2008.00965.x

Suzuki, M., Sato, S., Arai, Y., Shinohara, T., Tanaka, S., Greall, J.M., Hattori, N., Shiota, N. (2007). A new class of tissue-specifically methylated regions involving entire CpG islands in the mouse. *Genes to Cells*. 12, 1305–1314. DOI: 10.1111/j.1365-2443.2007.01136.x

Weinhofer, I., Hehenberger, E., Roszak, P., Hennig, L., Kohler, C. (2010). H3K27me3 Profiling of the Endosperm Implies Exclusion of Polycomb Group Protein Targeting by DNA Methylation. *PLoS Genet*. 6(10), e1001152. doi:10.1371/journal.pgen.1001152

Yan, Y., Shen, L., Chen, Y., Bao, S., Thong, Z., Yu, H. (2014). A MYB-Domain Protein EFM Mediates Flowering Responses to Environmental Cues in Arabidopsis. *Developmental Cell*. 30, 437–448

Zemach, A., Kim, M.Y., Silva, P., Rodrigues, J.A., Dotson, B., Brooks, M.D., Zilberman, D. (2010). Local DNA hypomethylation activates genes in rice endosperm. *Proc. Natl. Acad. Sci*. 107(43), 18729–18734. Doi: 10.1073/pnas.1009695107Zhang, H., Zhu, J-K. (2011) RNA-directed DNA methylation. *Curr. Opin. Plant Biol.* 14, 142-147.

Zhang, L., Peng, Y., Wei, X., Dai, Y., Yuan, D., Lu, Y., Pan, Y., Zhu, Z. (2014). Small RNAs as important regulators for the hybrid vigour of super-hybrid rice. *Journal of Experimental Botany*. doi:10.1093/jxb/eru337

Zheng, X., Zhu, J., Kapoor, A., Zhu, J.-K. (2007). Role of Arabidopsis AGO6 in siRNA accumulation, DNA methylation and transcriptional gene silencing. *The EMBO Journal*. 26, 1691–1701

Zheng, B., Chen, X. (2011). Dynamics of histone H3 lysine 27 trimethylation in plant development. *Current Opinion in Plant Biology*. 14:123–129
